# Dose uniformity of budesonide Easyhaler® under simulated real-life conditions and with low inspiration flow rates

**DOI:** 10.1177/1479972317745733

**Published:** 2017-12-07

**Authors:** Jussi Haikarainen, Paula Rytilä, Sirkku Roos, Sirpa Metsärinne, Anita Happonen

**Affiliations:** 1 Orion Corporation, Orion Pharma, Espoo, Finland; 2 Medfiles Ltd, Kuopio, Finland; 3 Orion Corporation, Orion Pharma, Kuopio, Finland

**Keywords:** Asthma, budesonide, dose uniformity, dry powder inhaler, Easyhaler®

## Abstract

Budesonide Easyhaler® multidose dry powder inhaler is approved for the treatment of asthma. Objectives were to determine the delivered dose (DD) uniformity of budesonide Easyhaler® in simulated real-world conditions and with different inspiration flow rates (IFRs). Three dose delivery studies were performed using 100, 200, and 400 µg/dose strengths of budesonide. Dose uniformity was assessed during in-use periods of 4–6 months after exposure to high temperature (30°C) and humidity (60% relative humidity) and after dropping and vibration testing. The influence of various IFRs (31, 43, and 54 L/min) on the DD was also investigated. Acceptable dose uniformity was declared when mean DD were within 80–120% of expected dose; all data reported descriptively. DD was constant (range: 93–109% of expected dose) at all in-use periods and after exposure to high temperature and humidity for a duration of up to 6 months. DD post-dropping and -vibration were unaffected (range 98–105% of expected dose). Similarly, DD was constant and within 10% of expected dose across all IFRs. Results indicate that budesonide Easyhaler® delivers consistently accurate doses in various real-life conditions. Budesonide Easyhaler® can be expected to consistently deliver a uniform dose and improve asthma control regardless of high temperature and humidity or varying IFR.

## Introduction

Asthma and chronic obstructive pulmonary disease (COPD) are highly prevalent chronic respiratory diseases and leading causes of morbidity and mortality worldwide.^1–3^ Inhalers are the mainstay device for therapeutic drug delivery in respiratory diseases^4^ and inhaled corticosteroids (ICS) are the first choice for an anti-inflammatory treatment.^3,5^ International guidelines recommend that asthma treatment follows a stepwise approach with an initial introduction of low dose ICS advised for all patients with persistent asthma.^6^

There are over 20 devices available for therapeutic administration in patients with asthma and COPD.^7^ The effectiveness of inhalers can be influenced by several factors including, ease of use, age, patient education, severity of disease, type of inhaler used, and inhalation technique.^8–11^ Unlike the traditional pressurized metered dose inhalers (pMDIs), dry powder inhalers (DPIs) are breath actuated, removing the difficulty of coordinating actuation with inhalation, often experienced using pMDIs.^11^ However, for some DPIs, release of the emitted dose can be inconsistent.^12^ DPIs require a sufficient degree of inspiratory flow to disaggregate the formulation and to deliver the whole dose accurately, an important consideration when treating patients with severe airway obstruction.^7^

Robustness of inhaler performance and functioning consistently under different conditions are key requirements for DPIs in enabling the successful management of asthma and COPD. Storage conditions (such as temperature and humidity^11^), device handling (e.g. dropping or shaking^11^), and variation in inspiration flow rates (IFRs)^13^ are all potential factors which may affect uniformity of the delivered dose (DD). The effectiveness of an inhaled treatment is dependent on the inhalation flow rates that patients can achieve, which is determined by the internal resistance to airflow inside the inhalation channel of the device.^14^

**Figure 1.**
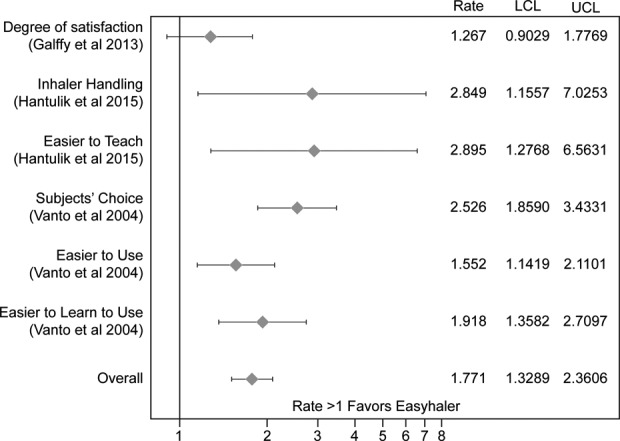
Meta-analysis of Easyhaler® preference in pediatric patients compared with other inhalers. Ratios and respective confidence intervals of preference for Galffy and Vanto studies as well as odds ratios and respective confidence intervals for Hantulik study were calculated. The overall estimate was calculated using the method of weighted least squares. The weights for individual items were the inverse for each single item.^21^ LCL: lower confidence interval; UCL: upper confidence interval.

Budesonide Easyhaler® (Orion Corporation, Orion Pharma, Espoo, Finland) is a multidose DPI, indicated for the treatment of patients with mild, moderate, and severe asthma and approved in many European countries. The available dose strengths are 100, 200, and 400 µg/dose. The maintenance dose in adults is 100–400 µg twice-daily, although the 200–400 µg doses are indicated for once-daily dosing in adults and children >12 years of age, and the 100 and 200 µg dose strengths are indicated for twice-daily delivery in children 6 years of age and above.^15^ Easyhaler® has been designed to be simple to use and practical, delivering accurate doses, as demonstrated in a number of in vitro and in vivo studies of flow rate dependency.^16^ Patient preferences (based on studies in children and adults) demonstrate Easyhaler® as being easier to teach, learn, and use, coupled with a greater degree of user satisfaction versus comparators.^17–19^ Accordingly, Easyhaler® was also favored over Turbuhaler® (AstraZeneca Plc, Gothenburg, Sweden) by the majority of patients in a randomized, double-blind, double-dummy, parallel-group study in asthmatic children.^20^ A recent meta-analysis assessing patient preferences and ease of use confirmed the findings from Vanto et al., overall rate ratio of 1.77 (confidence intervals: 1.33–2.36; Figure 1). Results from four pivotal pharmacokinetic studies showed that Easyhaler® demonstrated consistent fine particle dose across a wide range of IFRs with high lung deposition,^22^ supporting previous in vitro data which compared Easyhaler® with two comparator devices.^11^ In a retrospective, matched-cohort study of 1958 children and adults with asthma, patients who switched to Easyhaler® from other devices were significantly more likely to achieve overall asthma control and had lower costs associated with short-acting β_2_ agonist consumption and consultations, compared with patients who remained on an ICS device other than Easyhaler®.^23^

Here, we report the findings of three distinct studies, which aimed to establish the DD uniformity of the budesonide Easyhaler® in simulated real-world conditions, modeled using a variety of in vitro tests.

## Methods

### Study design

Three distinct dose delivery studies were conducted to assess the in vitro uniformity of budesonide Easyhaler® dose delivery in various simulated real-world conditions: first, at high temperature and humidity relative to ambient storage conditions, fixed over a 6-month period; second, mechanical stress (dropping and vibrations assays); and third, variation in IFRs. The following strengths of budesonide Easyhaler® were used in all assays: 100, 200, and 400 µg/dose. For all the dose delivery studies, drug doses were collected in accordance with the in vitro testing of DPIs established by the European Pharmacopoeia.^24^

### Dose delivery

Four liter of air was drawn through the inhaler at a flow rate corresponding to 4 kPa pressure drop across the device, as described in European Pharmacopoeia 8.0^25^. The inhaler was connected to the inlet of the apparatus using a mouthpiece adaptor that ensures a good seal, and the dose was collected in a sample collection tube. Collected budesonide was dissolved with 68:32 (v/v) water–acetonitrile and analyzed by high-performance liquid chromatography. Chromatographic separations were carried out on a Novapak RP-18 (4 µm, 3.9 × 150 mm^2^) analytical column. The mobile phase was a phosphate buffer solution: 4.2% m/V phosphoric acid solution, 0.3% m/V sodium dihydrogen phosphate buffer solution (1:9, v/v), adjusted to pH 3.2 with 5 M sodium hydroxide and acetonitrile (68:32, v/v). The mobile phase was delivered at a flow rate of 1.5 mL/min and the injection volume was 25 µL. Ultraviolet detection at 240 nm and run time 1.5 × retention time of budesonide were used. Calibration curves were linear over the budesonide range of 0.1–125 µg/mL (*r* ^2^ = 1.000). Recovery assays displayed an accuracy of 100 ± 2% across the range of budesonide concentrations tested (0.1–125 µg/mL). The quantitation limit of budesonide was 0.1 µg/mL (0.05% relative to the 200 μg/dose). The method also showed good repeatability and intermediate precision, as assessed by testing the inhalation powder samples on the same day and across 3 days, respectively. Specification limits of 80–120% (80–120, 160–240, and 320–480 µg/dose) were applied when assessing the mean budesonide DD (for the 100, 200 and 400 μg/doses, respectively), in accordance with the limits set by European Pharmacopoeia.^24^

### The effect of high temperature and humidity on dose uniformity

**Figure 2.**
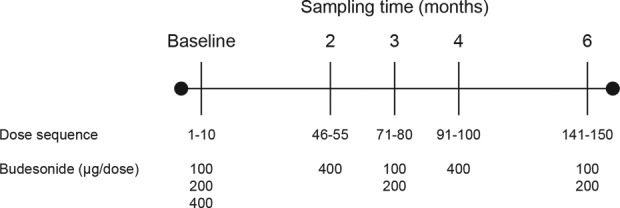
Schematic representation of the daily actuation, dosing sequence, and sampling times for assessing dose uniformity under warm and humid conditions.

Budesonide Easyhaler®s were stored after opening the laminate pouch in a controlled environment room set to 30°C and 60% relative humidity. Figure 2 describes the daily actuation, dosing sequence, and sampling times used over a 6-month period. A total of eight devices were tested (two devices for 100 and 400 µg/dose strengths from one batch and four devices for 200 µg/dose strength from two batches). Five doses were measured for each inhaler used, and the mean DD calculated.

### The effect of mechanical stress on dose uniformity: Dropping

Baseline was determined by analyzing the DD of the first five doses. After that the inhaler was dropped from a 1 m high platform on to a hard wooden surface, and the DD of the next five doses was analyzed. A total of 11 devices were tested (four devices for each 100 and 400 µg strength of budesonide and three devices for 200 µg; one batch/strength of budesonide was used). Five doses were measured for each inhaler used, and the mean DD calculated.

### The effect of mechanical stress on dose uniformity: Vibration

Baseline was determined by analyzing the DD of the first five doses. DD of the next five dose strengths was analyzed following British standard vibration test.^26^ A total of nine devices were tested (three devices and a single batch for each dose strength). Five doses were measured for each inhaler used, and the mean DD calculated.

### The effect of varying IFR on dose uniformity

DD were measured over a range of IFRs (2, 4, and 6 kPa, comparable to flow rates of 31, 43, and 54 L/min, respectively) under ambient laboratory conditions (room temperature and relative humidity), for each strength of budesonide. For each flow rate, 4 L of air was drawn through the inhaler (according to Ph. Eur. method^24^). A total of 27 devices were tested (three devices for each flow rate and strength of budesonide; a single batch was used for each budesonide strength). Ten doses were measured for each inhaler used, and the mean DD calculated.

### Data analysis

Descriptive statistics, mean, and standard deviations were used to describe all data.

## Results

Overall, 55 devices were used. Five batches of 200 µg/dose and four batches of both 100 and 400 µg/doses of budesonide were tested for DD, with no noticeable batch-to-batch variation observed.

### The effect of high temperature and humidity on dose uniformity

**Figure 3.**
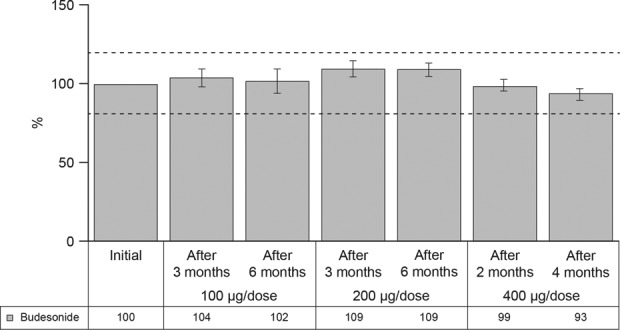
DD during simulation of patient use under high temperature (30°C) and humidity (60% relative humidity). Dashed lines represent the upper and lower specification limits (%). DD: delivered dose.

The DD of budesonide was consistent following exposure to 30°C and 60% relative humidity, for up to 6 months (Figure 3). All mean DD values remained within the preselected specification limits for the duration of the study. All DDs were within 10% of the expected labeled strength (range, 93–109%), for all time points assessed.

### The effect of dropping and vibration testing on dose uniformity

**Figure 4.**
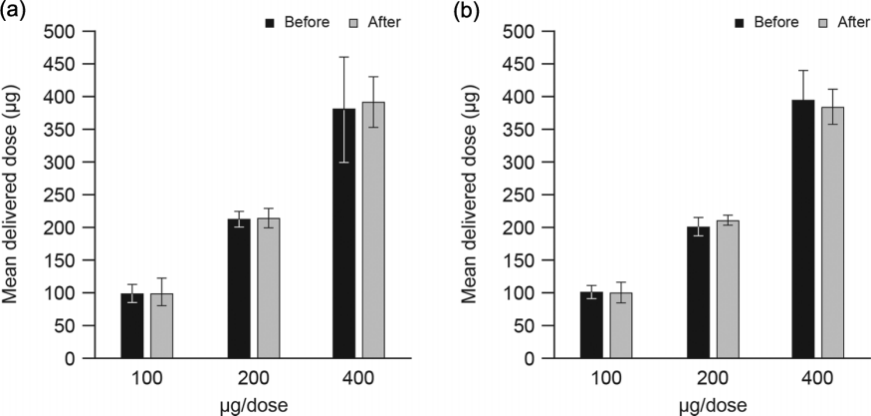
DD of budesonide using Easyhaler® before and after: (a) dropping tests and (b) vibration tests. Data presented as mean ± standard deviation. DD: delivered dose.

The DD of budesonide was consistent with baseline assessments at all prespecified doses after dropping and vibration (Figure 4). All mean DDs remained within the preselected specification limits. All DDs post-dropping were <4% from the expected labeled strength (range, 101–103%). Similarly, all DDs post-vibration were close to the expected labeled strength (range, 98–105%). No device breakages were detected and functionality remained unaffected after the dropping and vibration tests.

### The effect of varying IFRs on dose uniformity

**Figure 5.**
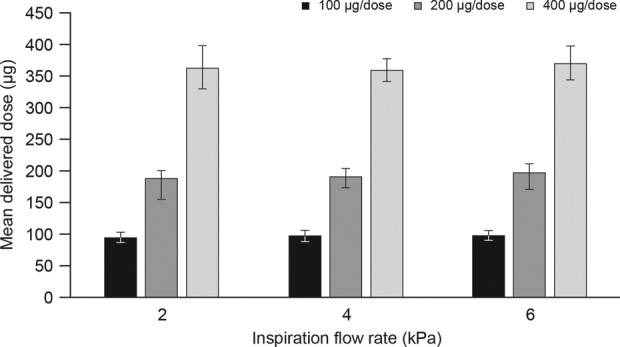
DD of budesonide using Easyhaler® following actuation at different IFRs. Data presented as mean ± standard deviation. IFR: inspiration flow rate; DD: delivered dose.

The DD of budesonide remained consistent across the three IFRs applied (Figure 5). The mean DD varied within 3, 9, and 11 µg range for the 100, 200, and 400 µg/dose strengths, respectively. All DDs were within 10% of the expected labeled strength (range, 90–100%) for each flow rate tested.

## Discussion

The in vitro tests described here demonstrate that budesonide Easyhaler® delivers a highly uniform dose under conditions designed to simulate real-world conditions.

In real-life use, DPIs may periodically be exposed to humid conditions that may affect aerosol characteristics and lung deposition. Here, DD was consistent in all tests performed, indicating that budesonide Easyhaler® delivers complete and accurate doses regardless of storage temperature and humidity, improper handling (e.g. dropping), or applied IFR, under controlled laboratory conditions which are likely to reflect real-world use. These results demonstrate the robustness of budesonide Easyhaler® (through consistency of DD) under high temperature and humidity, relative to ambient storage conditions. The uniform DD was also maintained for all strengths of budesonide after dropping. In contrast, another widely available inhaler platform (Duoresp Spiromax; Teva UK Ltd, Castleford, UK) has been shown to be more sensitive to the effects of dropping as an 80% increase in first dose was recorded after dropping tests.^27^

Dose accuracy and consistency of salbutamol delivery using Easyhaler® is less sensitive to flow rates than other multidose DPIs.^28^ Here, a highly uniform DD for all strengths of budesonide (mean DD 90–110% of the expected labeled dose across all simulated conditions) was observed irrespective of IFR, supporting previous data showing that the Easyhaler® DD is independent of IFR.^16,28^ Accordingly, variations in IFR also had a minimal effect on Easyhaler® DD for all doses used, with observed differences in mean DD <5% across all IFRs tested.

In vitro dose delivery studies have limitations. Here, the main limitation was the performance of each study under laboratory conditions. Although designed to evaluate the impact of possible factors which arise in everyday use (such as accidently dropping the inhaler onto a hard floor), these studies cannot mimic all conceivable real-life scenarios. This is pertinent to the tests performed at high temperature and humidity (relative to ambient storage conditions); in our study, consistent DD within 10% of the expected label strength was achieved at all time points, but further tests (e.g. of performance of Easyhaler® in freezing temperatures or at different relative humidity) would be required to confirm dose consistency in a more comprehensive range of conditions, which would be relevant in different geographical regions, for example. Also, the findings cannot account for more than one real-life scenario occurring simultaneously nor for different severity of certain conditions (e.g. the inhaler dropping from a greater height or on to a different type of surface). Finally, although standard laboratory procedures were performed, a lack of a reference medicinal product or other controls means comparisons with other inhalers cannot be drawn.

Overall, the data presented suggest that reliability of budesonide Easyhaler® may be assured in a range of conditions designed to mimic real-world use. Together with previous findings that Easyhaler® is easy-to-use,^17–19^ this evidence is likely to be of benefit to clinicians who require inhalers that perform reliably in patients with severe airway obstruction (Easyhaler® also offers the option of several drug substances administered via the same device). These data also add support to the current evidence demonstrating consistent DD, in patients with asthma who have used Easyhaler® devices.^11,22^

## Conclusions

These results indicate that budesonide Easyhaler® delivers consistently accurate doses throughout the inhaler life under simulated real-life conditions. Treatment efficacy of budesonide is unlikely to be affected by variations in IFR, by dropping the inhaler, or by using it where temperature or humidity is high. Easyhaler® is preferable to other inhaler devices for patients with airway disease,^10, 11, 23^ potentially influencing disease management through increased compliance to treatment and adherence. Easyhaler® applied to real-world use in the treatment of patients with obstructive airway diseases like COPD and asthma can be expected to consistently deliver a uniform dose and improve disease control.
